# The Effectiveness of Facet Joint Injection with Steroid and Botulinum Toxin in Severe Lumbar Central Spinal Stenosis: A Randomized Controlled Trial

**DOI:** 10.3390/toxins15010011

**Published:** 2022-12-23

**Authors:** Sang Hoon Lee, Hyun Hee Choi, Min Cheol Chang

**Affiliations:** 1 Department of Radiology, Madi Pain Management Center, Jeonju 54969, Republic of Korea; 2 Madi Research and Development Center, Jeonju 54969, Republic of Korea; 3 Department of Rehabilitation Medicine, College of Medicine, Yeungnam University, Daegu 42415, Republic of Korea

**Keywords:** lumbar spine, spinal stenosis, pain, radicular pain, facet joint, epidural injection, steroid, botulinum toxin

## Abstract

Lumbar central spinal stenosis (LCSS) is a common disorder that causes disability and pain in the elderly. It causes pain in the radicular leg. Recently, transforaminal epidural steroid injection (TFESI) has been widely used to control radicular leg pain caused by LCSS. However, in cases of severe LCSS, drugs injected using TFESI cannot spread into the spinal canal and would have less therapeutic effects than in mild LCSS. To compensate for this limitation of TFESI, we injected steroids and botulinum toxin type A into the bilateral facet joints, evaluated their effects, and compared them with those of TFESI. One hundred patients with severe LCSS were included in the study and randomly allocated to either the facet injection (FI) or TFESI group. For 50 patients in the FI group, 30 mg (40 mg/mL) of triamcinolone with 50 IU of botulinum toxin type A mixed with a 1 mL solution of 100 mL of 50% dextrose water and 30 mL of 4% lidocaine were administered into the bilateral facet joints under fluoroscopy. For 50 patients in the TFESI group, 30 mg (40 mg/mL) of triamcinolone with 0.8 mL of 2% lidocaine and 2.5 mL of 50% dextrose water was injected bilaterally under fluoroscopy. Radicular leg pain (measured with a numeric rating scale) and pain-related disability (measured with the modified Oswestry Disability Index) due to severe LCSS were significantly reduced after facet joint injection. The therapeutic effects were greater after facet joint injection than after bilateral TFESI. The injection of a mixed solution of steroids and botulinum toxin type A into the bilateral facet joints would be a beneficial therapeutic option in patients with severe LCSS.

## 1. Introduction

The prevalence of symptomatic lumbar central spinal stenosis (LCSS) is approximately 27% in the general population [1]. LCSS is caused by the innate or acquired narrowing of the spinal canal. It results in encroachment of the lumbosacral nerve roots by the surrounding bone and soft tissues, which can cause radicular leg pain during walking or long standing [1,2]. For the management of pain from LCSS, various therapeutic methods, such as analgesic medications, physical therapy, exercise, and injection procedures, can be applied [3,4,5].

Epidural steroid injections are commonly administered to patients with LCSS [6,7,8,9]. Injected steroids can reduce nerve root inflammation induced by encroached nerve roots at the narrowed spinal canal [4]. For epidural steroid injection, two methods can be used: interlaminar epidural steroid injection and transforaminal epidural steroid injection (TFESI) [10]. Because mechanical and inflammatory reactions playing important roles in provoking radicular leg pain mainly occur between the intervertebral disc, posterior longitudinal ligament, and nerve root, steroids would be better to be administered into the ventral epidural space [11,12]. Interlaminar epidural steroid injections deliver steroids into the posterior epidural spaces, with the expectation that the injected drugs can spread to the ventral spaces afterward [10]. In contrast, TFESI can directly deliver steroids into the ventral epidural space [10]. Previous studies have reported that TFESI showed a better pain-controlling effect in patients with LCSS than interlaminar epidural steroid injection [13,14]. However, in cases of severe LCSS, drugs injected using TFESI cannot spread into the spinal canal and would have less therapeutic effects than in mild LCSS.

Recently, some previous studies reported that steroid injection into the bilateral facet joints had a better therapeutic effect in LCSS through the epidural spread of the injected drug from the facet joint space compared with that of interlaminar epidural steroid injection [15,16]. In addition, botulinum toxin has been reported to exert an analgesic effect on neuropathic and nociceptive pain by inhibiting the release of neuropeptides that sensitize nociceptors [17,18].

The present study evaluated the effect of steroid and botulinum toxin type A injections into the bilateral facet joints in severe LCSS and compared their effects with that of bilateral TFESI. The main outcomes of our study were changes in leg pain and functional disability, which were measured by the numeric rating scale (NRS) and the modified Oswestry Disability Index (MODI), respectively.

## 2. Results

None of the patients dropped out during the follow-up period. No adverse events were observed in the FI or TFESI groups. There were also no significant differences in demographic data between the FI and TFESI groups (Table 1, *p* > 0.05). In addition, there was no significant difference in the number of injections between the two groups (*p* = 0.608; the mean number of injections in the facet injection (FI) group = 2.3 ± 0.9; the mean number of injections in the TFESI group = 2.3 ± 0.6).

**Figure 1:**
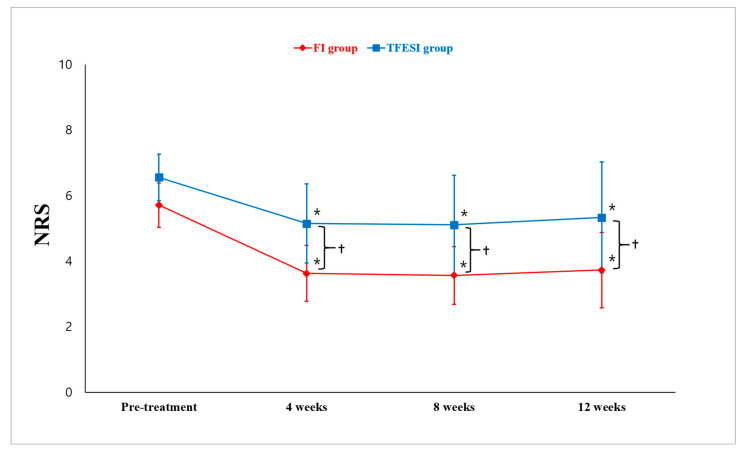
Change in numeric rating scale (NRS). In terms of pretreatment NRS scores, the facet injection (FI) and transforaminal epidural steroid injection (TFESI) groups showed significant decreases in scores at 4, 8, and 12 weeks after injection. In addition, 4, 8, and 12 weeks after the injections, the NRS score was significantly lower in the FI group than in the TFESI group. * *p* < 0.05: Intragroup comparison between pretreatment and 4, 8, and 12 weeks post-treatment (repeated-measure 1-factor analysis). † *p* < 0.05: Intergroup comparison at each evaluation time (repeated-measure 2-factor analysis).

Regarding the NRS changes after treatment, the mean NRS score in the FI group decreased after treatment. The pretreatment NRS was 5.7 ± 0.7. At 4, 8, and 12 weeks after injection, the mean NRS was 3.6 ± 0.8, 3.6 ± 0.9, and 3.7 ± 1.2, respectively (Figure 1). In the TFESI group, the mean NRS decreased from 6.6 ± 0.7 at pretreatment to 5.1 ± 1.2 at 4 weeks, 5.1 ± 1.5 at 8 weeks, and 5.3 ± 1.7 at 12 weeks after injection. The NRS scores for each group were significantly different over time (*p* < 0.001) (Figure 1). In both groups, the scores at 4, 8, and 12 weeks were significantly lower than the pretreatment scores (*p* < 0.001). Reductions in NRS scores over time were significantly larger in the FI group than in the TFESI group (*p* < 0.001) (Figure 1). In addition, the NRS scores from pretreatment to each evaluation time point were significantly more reduced in the FI group than in the TFESI group (*p* < 0.001).

**Figure 2:**
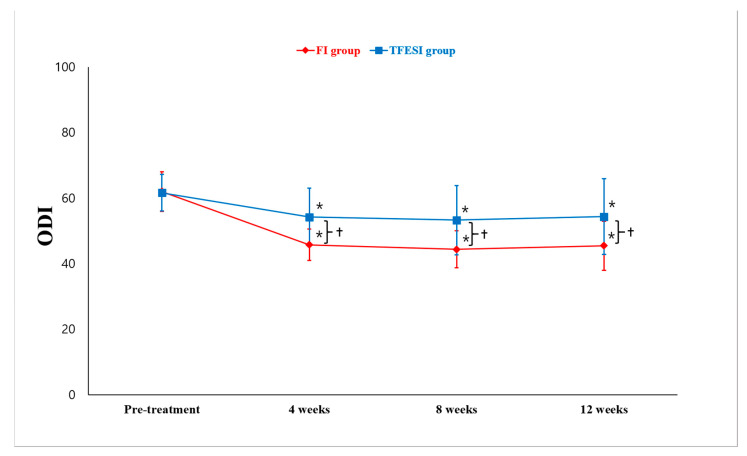
Change in modified Oswestry Disability Index (MODI). In terms of pretreatment MODI scores, the facet injection (FI) and transforaminal epidural steroid injection (TFESI) groups showed a significant decrease in scores at 4, 8, and 12 weeks after injection. In addition, 4, 8, and 12 weeks after the injections, the MODI score was significantly lower in the FI group than in the TFESI group. * *p* < 0.05: Intragroup comparison between pretreatment and 4, 8, and 12 weeks post-treatment (repeated-measure 1-factor analysis). † *p* < 0.05: Intergroup comparison at each evaluation time (repeated-measure 2-factor analysis).

Regarding the MODI changes after treatment, the mean MODI in the FI group decreased after treatment. The pretreatment MODI was 62.0 ± 6.1. At 4, 8, and 12 weeks after injection, the mean MODI was 45.7 ± 4.8, 44.3 ± 5.7, and 45.4 ± 7.5, respectively (Figure 2). In the TFESI group, the mean MODI decreased from 61.6 ± 5.6 pretreatment to 54.2 ± 8.9 at 4 weeks, 53.2 ± 10.6 at 8 weeks, and 54.3 ± 11.6 at 12 weeks after injection. The MODI scores for each group were significantly different over time (*p* < 0.001) (Figure 2). In both groups, the scores at 4, 8, and 12 weeks were significantly lower than the pretreatment scores (*p* < 0.001). The reduction in the ODI scores over time was significantly larger in the FI group than in the TFESI group (*p* < 0.001) (Figure 2). In addition, the MODI scores from pretreatment to each evaluation time point were significantly reduced in the FI group than in the TFESI group (*p* < 0.001).

## 3. Discussion

In our study, we found that the injection of steroids and botulinum toxin type A into the bilateral facet joints had a positive therapeutic effect for controlling radicular leg pain and improving functional disability due to severe LCSS. This effect was greater than that of bilateral TFESI.

Facet joint injection is usually performed to control facet-origin axial back pain [19]. However, recently, it was suggested that facet joint injection can be an alternative treatment for lumbar radicular pain following LCSS via the epidural spread of the injected drug from the lumbar facet joint space [15,16]. In patients with LCSS, facet degeneration is known to occur in combination with disc protrusion and ligamentum flavum hypertrophy [20]. In the degenerated facet joint, the facet capsule is likely to rupture and tear [21]. Therefore, the drug injected into the facet joint space would more easily spread into the epidural space in severe LCSS.

Compared with epidural steroid injection, facet joint injection has the advantage of being a safe and less invasive technique, because it does not directly puncture the epidural space or spinal radicular artery [22]. In contrast, epidural steroid injection can cause rare but major complications, including spinal infarction and epidural hematoma, through the inadvertent puncture of the radicular artery supplying the spinal cord and rupture of the venous plexus in the epidural space [23,24,25]. Therefore, bilateral facet joint injection seems to be a safe procedure that can control radicular pain from LCSS with better effects than epidural steroid injections. However, the injection of steroids into facet joints is reported to have a limited long-term effect on chronic spinal pain [26]. We think that the injection of botulinum toxin A mixed with steroids might be helpful for prolonging the therapeutic effect.

Botulinum toxin type A is known to have analgesic effects [17,18]. It can inhibit the release of various neurotransmitters and neuropeptides that sensitize nociceptors, such as glutamate, calcitonin-gene-related peptides, and substance P [17,18,27]. Therefore, botulinum toxin type A can reduce pain and prevent nociceptive sensitization. The inhibition of nociceptor sensitization interferes with the pathogenic mechanism of pain amplification. Accordingly, we believe that the injection of botulinum toxin type A in our patients could have contributed to the reduction in radicular pain due to LCSS. In addition, we think that the spread of botulinum toxin type A into the paraspinal muscle from the inferior facet recess might relax muscle contraction and reduce the degree of the mechanical compression of the nerve roots.

In patients with LCSS, mechanical compression or chemical irritation around the nerve root contributes to radicular leg pain [4]. Bilateral facet joint injection delivers injected drugs into the bilateral epidural space, in which nerve roots that cause radicular pain due to LCSS are located via epidural spread from the facet joint space [15,16]. In contrast, in severe LCSS, TFESI can be limited to the delivery of the injected drugs into the epidural space due to mechanical obstruction by hypertrophied bony structures, facet joints, and ligaments.

In conclusion, we showed that radicular leg pain and pain-related disability due to severe LCSS were significantly reduced after the injection of a mixed solution of steroids and botulinum toxin type A into the bilateral facet joints. Furthermore, it had a better therapeutic effect than bilateral TFESI. We believe that the injection of steroids and botulinum toxin type A into the bilateral facet joints is a feasible therapeutic option in patients with severe LCSS. Our study had some limitations. First, a long-term follow-up was not conducted. Second, the effects of facet injection and TFESI were not compared with those of the placebo procedure. Third, our study included a relatively small number of participants. Fourth, the required sample size could not be calculated because there was no previous research that could be used as a reference for our study. Further studies addressing these limitations are warranted.

## 4. Materials and Methods

**Figure 3:**
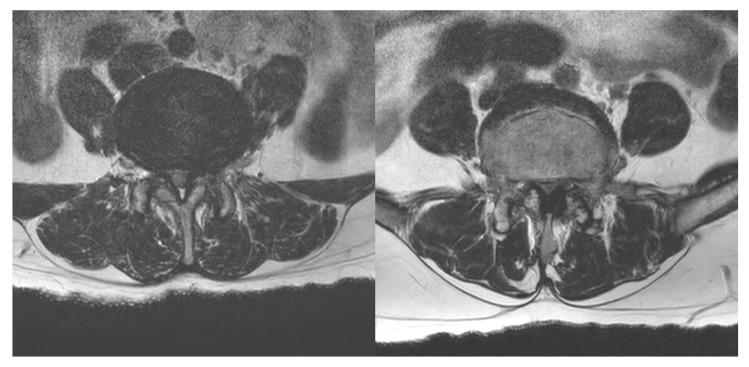
Severe (**left**) and extreme (**right**) lumbar canal spinal stenosis on T2-axial magnetic resonance imaging.

This prospective study was conducted at a single outpatient pain clinic. We prospectively recruited 100 consecutive patients with lower leg pain due to LCSS according to the following inclusion criteria: (1) age between 20 and 79 years; (2) ≥3 months history of lower leg pain due to LCSS, characterized by bilateral or unilateral leg pain in a diffuse distribution during walking or prolonged standing with relief by sitting or leaning forward; (3) pain intensity of >5 on an NRS (0 = no pain, 10 = the worst pain); and (4) severe or extreme LCSS on T2-axial magnetic resonance imaging. LCSS severity was determined based on a previous study by Schizas et al. [28]. In severe stenosis, rootlets and cerebrospinal fluid were not visible, but epidural fat was visible posteriorly; in extreme stenosis, rootlets, cerebrospinal fluid, and epidural fat were not visible (Figure 3). The exclusion criteria were as follows: (1) the presence of herniated lumbar disc, myelopathy, or infection on the spine; (2) the presence of more than one segment of severe or extreme LCSS; (3) previous history of spinal surgery, such as laminectomy or lumbar fusion; (4) coagulation disorder; (5) osteoporotic compression fracture; (6) multiple-level spinal stenosis; (7) vascular stenosis in the lower extremity; and (8) the severe deterioration of general condition. The institutional review board of the university hospital approved the study, and all the patients provided their signed informed consent.

The recruited patients were randomly allocated to two groups. Randomization was performed using a table of random numbers. The patients were allocated using sealed, opaque envelopes containing the randomization. Fifty patients were classified into the FI group, in which steroids and botulinum toxin type A were injected into the corresponding bilateral facet joints, and the other 50 patients were classified into the TFESI group, in which TFESI was conducted bilaterally on the corresponding level. Facet joint injection and TFESI were performed up to three times at intervals of 2 weeks until radicular leg pain was reduced by ≥60% of pain at pretreatment.

### 4.1. Facet Joint Injection Procedure

**Figure 4:**
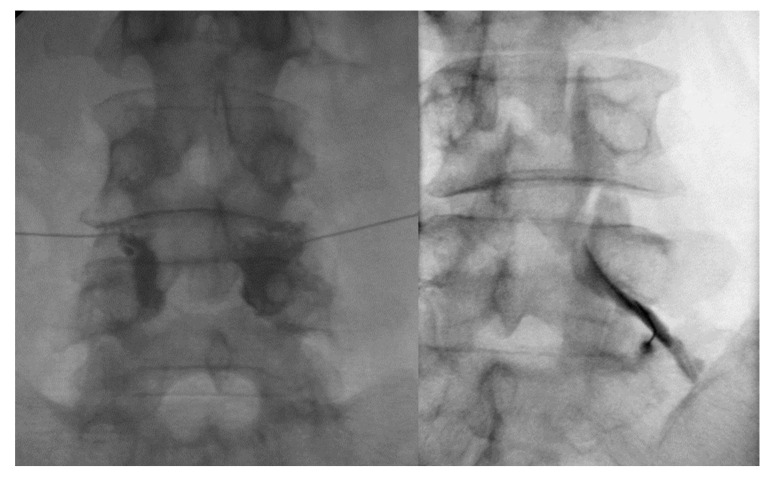
Fluoroscopy-guided facet joint injection (**left**) and transforaminal epidural steroid injection (**right**).

The patients were placed prone, and the fluoroscopic tube (Ziehm, Nuremberg, Germany) was angled cephalad and rotated until it was tangent to the lumbar facet joint space. Under the guidance of fluoroscopy, the tip of a 25-gauge, 90 mm spinal needle with a bend at the tip was positioned within the inferior facet recess of the targeted lumbar facet joint. After confirming intra-articular access by injecting 0.3 mL of contrast into the lumbar facet joint space (Figure 4, left), 30 mg (40 mg/mL) of triamcinolone with 50 IU of botulinum toxin type A mixed with a 1 mL solution of 100 mL of 50% dextrose water and 30 mL of 4% lidocaine were administered. During the follow-up period after the first injection, all of the recruited patients received no additional oral medication or physical therapy.

### 4.2. TFESI Procedure

All the injections were performed by a single interventionist with 20 years of experience in pain intervention. A strict aseptic technique was applied to all the procedures. The patients were placed prone, and the fluoroscopic tube (Ziehm, Nuremberg, Germany) was rotated obliquely to the ipsilateral oblique ankle with respect to the suspected nerve root. Under the guidance of fluoroscopy, the tip of a 25-gauge, 90 mm spinal needle with a bend at the tip was positioned between the lateral vertebral body and the 6 o’clock position below the pedicle. Lateral fluoroscopic imaging demonstrated a needle tip between the spinal laminar margin and posterior vertebral body. Under anteroposterior fluoroscopy, approximately 1 mL of non-ionic contrast material was injected to confirm the absence of vascular uptake and the spread of contrast into the foramen (Figure 4, right). Subsequently, 30 mg (40 mg/mL) of triamcinolone with 0.8 mL of 2% lidocaine and 2.5 mL of 50% dextrose water was injected. TFESI was conducted sequentially on the right and left neural foramina.

### 4.3. Outcomes

The same investigator, who was blinded to the patients’ grouping and did not participate in the treatment, assessed the degree of leg pain due to LCSS using NRS and the functional disabilities using the MODI at pretreatment and all follow-up assessments. Follow-up assessments were conducted 4, 8, and 12 weeks after the first injection.

### 4.4. Statistical Analysis

Data were analyzed using the Statistical Package for Social Science (SPSS, v. 32.0, SPSS Inc., Chicago, Ill., USA). Demographic data and the number of injections were compared between the TFESI and FI groups using the independent *t*-test and chi-square test. The changes in NRS and MODI scores in the TFESI and FI groups were evaluated using repeated-measure 1-factor analysis. Repeated-measure 2-factor analysis was used to compare the changes between the groups over time. Multiple comparisons were obtained following a contrast under Bonferroni correction. Statistical significance was set at *p* < 0.05.

## Figures and Tables

**Table 1:** Demographic data and baseline clinical data of the facet injection (FI) and transforaminal epidural steroid injection (TFESI) groups.

	FI Group	TFESI Group	*p* Value
Age, years	69.4 ± 7.5	69.2 ± 7.1	0.870
Sex (M:F), *n*	18:32	21:29	0.539
Level (L2–3:L3–4:L4–5), *n*	1:4:45	0:1:49	0.204
Pain duration, months	28.9 ± 17.3	28.4 ± 16.9	0.889
Stenosis grade (severe:extreme)	12:38	20:30	0.086
Initial NRS	6.5 ± 0.7	6.6 ± 0.7	0.564
Initial MODI	62.0 ± 6.1	61.6 ± 5.6	0.758

The values presented are numbers or the mean ± standard deviation. NRS, numeric rating scale; MODI, modified Oswestry Disability Index.

## Data Availability

The data presented in this study are available on request from the corresponding author.
